# Wiskott-Aldrich Syndrome With Severe Thrombocytopenia and Hemorrhagic Manifestations: A Case Report

**DOI:** 10.7759/cureus.111364

**Published:** 2026-06-23

**Authors:** Chaymae Cherrabi, Hassnae Tkak, Mohamed Bellaoui, Ayad Ghanam, Maria Rkain

**Affiliations:** 1 Department of Pediatric Services, Centre Hospitalier Universitaire Mohammed VI, Oujda, MAR; 2 Department of Pediatrics, Mohammed VI University Hospital, Oujda, MAR; 3 Genetics Unit, Faculty of Medicine and Pharmacy of Oujda, Mohammed I University of Oujda, Oujda, MAR; 4 Department of Pediatric Medicine, Centre Hospitalier Universitaire Mohammed VI, Oujda, MAR; 5 Faculty of Medicine and Pharmacy, Mohammed I University of Oujda, Oujda, MAR; 6 Department of Pediatric Gastroenterology, Centre Hospitalier Universitaire Mohammed VI, Oujda, MAR

**Keywords:** pediatric case report, primary immunodeficiency, thrombocytopenia intracranial hemorrhage, wiskott-aldrich syndrome, x-linked immunodeficiency

## Abstract

Wiskott-Aldrich syndrome (WAS) is a rare X-linked primary immunodeficiency characterized by the association of thrombocytopenia with microplatelets, eczema, and immune dysfunction, with a highly variable clinical presentation that may include severe hemorrhagic and infectious manifestations in early childhood. We report the case of an infant referred for evaluation of a hemorrhagic syndrome associated with eczema, in whom laboratory investigations revealed severe thrombocytopenia. The clinical course was complicated by a cerebral hemorrhage. The patient was managed with supportive measures, including intravenous immunoglobulin therapy and antibiotic prophylaxis. Despite treatment, thrombocytopenia persisted and required repeated platelet transfusions. Immunosuppressive therapy with corticosteroids and ciclosporin was introduced. This case highlights the importance of early recognition of WAS in infants presenting with thrombocytopenia and eczema, and emphasizes that management remains mainly supportive, while early evaluation for hematopoietic stem cell transplantation is essential to improve prognosis.

## Introduction

According to the most recent International Union of Immunological Societies (IUIS) classification of inborn errors of immunity, Wiskott-Aldrich syndrome (WAS) is categorized among combined immunodeficiencies with syndromic features [1]. It is a rare X-linked disorder characterized by microthrombocytopenia, eczema, and combined immunodeficiency. Beyond recurrent infections, immune dysregulation is a central aspect of the disease, with a high frequency of autoimmune and inflammatory manifestations that significantly contribute to morbidity and disease severity [2]. Clinical expression is highly heterogeneous, ranging from classical WAS to the milder X-linked thrombocytopenia (XLT) [3]. This variability reflects a clear genotype-phenotype correlation, where severe loss-of-function mutations are typically associated with the full clinical phenotype, whereas hypomorphic mutations result in attenuated disease presentations [2].

Although microthrombocytopenia is considered a hallmark of WAS, atypical presentations with normal platelet size have occasionally been reported and may delay diagnosis. We report a genetically confirmed case of WAS presenting with severe thrombocytopenia, absence of microplatelets, and intracranial hemorrhage, highlighting the importance of considering molecular testing even when classical hematological features are absent.

## Case presentation

A seven-month-old male infant, born to non-consanguineous parents (family history was initially considered negative), with an unremarkable neonatal history and incomplete immunization status, was referred to our department for the evaluation of a hemorrhagic syndrome characterized by recurrent bilateral epistaxis of moderate severity and repeated mucocutaneous bleeding episodes occurring in the setting of chronic eczema.

The patient had experienced chronic diarrhea since the first days of life, initially managed at the primary care level with symptomatic and dietary treatment without significant improvement. A pruritic erythematous and scaly skin eruption developed on day 15 of life, initially involving the flexural areas before progressively becoming generalized. The lesions had been treated for a prolonged period with topical corticosteroids. Petechiae were first noted during the first month of life, predominantly affecting areas of skin excoriation related to scratching.

Physical examination revealed severe failure to thrive, marked mucocutaneous pallor, and hepatosplenomegaly extending approximately 2 cm below the costal margin. Diffuse eczema consisting of widespread pruritic erythematous and scaly lesions was observed (Figure 1), together with cutaneous hemorrhagic manifestations in the form of petechiae (Figure 2).

**Figure 1:**
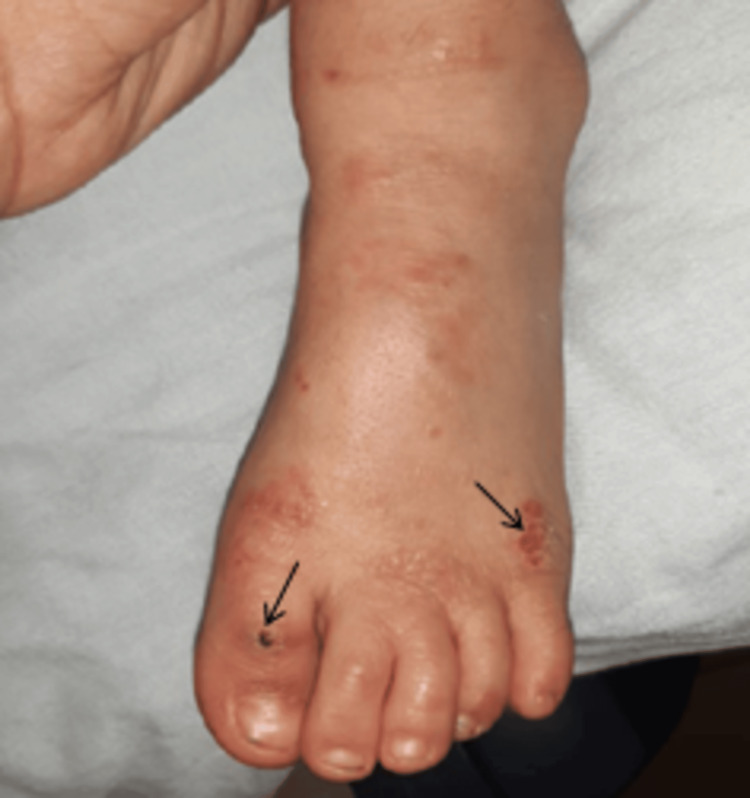
Eczematous skin lesions on the left foot Clinical photograph showing eczematous lesions (arrow) involving the left foot of the patient, consistent with the cutaneous manifestations of Wiskott-Aldrich syndrome.

**Figure 2:**
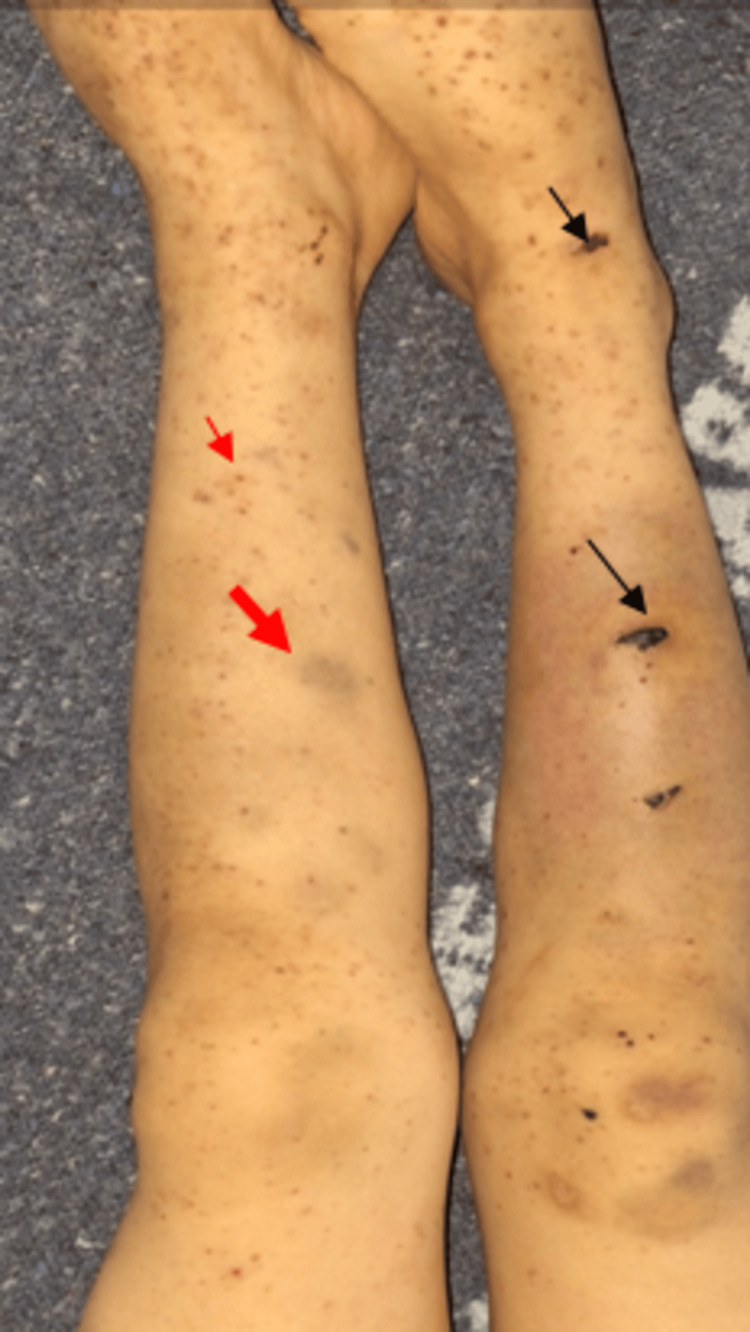
Eczematous lesions, petechiae, and ecchymoses on the lower limbs Clinical photograph of the lower limbs at four years of age showing eczematous lesions (black arrows) associated with petechiae and ecchymoses (red arrows), consistent with the cutaneous and hemorrhagic manifestations of Wiskott-Aldrich syndrome.

Laboratory investigations revealed severe normocytic hypochromic anemia with a hemoglobin concentration of 4.9 g/dL and a regenerative reticulocyte response, associated with severe peripheral thrombocytopenia (17,000/mm³) (Table 1). Iron studies showed low serum ferritin (20 ng/mL), suggestive of early iron deficiency. Peripheral blood smear examination confirmed thrombocytopenia with platelet anisocytosis, characterized by marked variation in platelet size without evidence of microplatelets. Bone marrow aspiration revealed a moderately cellular marrow with preserved myeloid maturation and 3% blasts. No megakaryocytes were observed, and there was no evidence of global marrow aplasia. The remainder of the hemostatic workup was within normal limits.

**Table 1:** Laboratory findings: complete blood count (CBC) results Hb: hemoglobin; Hct: hematocrit; MCV: mean corpuscular volume; MCH: mean corpuscular hemoglobin; MCHC: mean corpuscular hemoglobin concentration; PLT: platelets; MPV: mean platelet volume; WBC: white blood cell count; ANC: absolute neutrophil count

Parameters	Result	Reference range
HB	4.9 g/dl	13-18 g/dl
Hct	17.9%	40-54%
MCV	80 fL	80-98 fL
MCH	22.10 pg	27-32 pg
MCHC	27.4%	32-36%
PLT	17,000 cells/µl	150,000-400,000 cells/µl
MPV	8.3 fl	7-9 fl
WBC	22,270 cells/µl	4,000-10,000 cells/µl
ANC	6,730 cells/µl	1,500-7,000 cells/µl
Lymphocytes	9,390 cells/µl	1,000-4,000 cells/µl
Monocytes	3,020 cells/µl	200-800 cells/µl
Eosinophiles	2,970 cells/µl	0-500 cells/µl
Réticulocytes	125,400 cells/µl	20,000-80,000 cells/µl

Immunological evaluation revealed mildly elevated serum IgM levels, markedly increased total IgE concentrations exceeding 1,000 IU/mL, and elevated IgA and IgG levels (Table 2). The elevated IgA and IgG levels were interpreted as secondary to chronic antigenic stimulation related to recurrent infections and repeated skin superinfections. Functional antibody response testing was not available in our institution. Human immunodeficiency virus (HIV) serology was negative. Lymphocyte subset analysis demonstrated reduced T-cell counts, including both CD8+ and CD4+ populations, with an inverted CD4/CD8 ratio (0.75), reflecting an imbalance in T-cell subsets. A decrease in B lymphocytes (CD19+) was also observed, while natural killer (NK) cells (CD16+/CD56+) were relatively preserved (Table 2).

**Table 2:** Lymphocyte subset analysis and serum immunoglobulin profile Serum immunoglobulin profiling demonstrated decreased IgM levels, with elevated IgA and IgG concentrations, and markedly increased total IgE (>1,000 IU/mL), alongside lymphocyte subset analysis showing quantification of T lymphocytes (CD3+), T helper cells (CD4+), cytotoxic T cells (CD8+), B lymphocytes (CD19+), and natural killer (NK) cells.

Parameters	Result	Reference range
IgA	2.69 g/L	0.33-1.22 g/L
IgM	1.97 g/L	0.48-1.43 g/L
IgG	21.12 g/L	3.4-6.2 g/L
IgE	1,200 UI/ml	<60 UI/ml
Absolute CD19 cell count	1,040/mm³	390-1,400 cells/mm^3^
Absolute CD3 cell count	1,118/mm³	1,400-3,700 cells/mm^3^
Absolute CD8 count	581/mm³	490-1,300 cells/mm^3^
Absolute CD4 count	437/mm³	700-2,200 cells/mm^3^
NK	158/mm³	130-720 cells/mm^3^

Given the association of thrombocytopenia-related hemorrhagic manifestations, eczema, and recurrent infections, WAS was suspected. Genetic testing identified a pathogenic in-frame deletion in the WAS gene (NM_000377.3:c.1185_1190del), resulting in p.Pro403_Pro404del, thereby confirming the diagnosis.

The patient was started on cotrimoxazole prophylaxis (20 mg/kg every other day) and monthly intravenous immunoglobulin (IVIG) replacement therapy at a dose of 0.4 g/kg every 21 days. IVIG was administered as replacement/prophylactic therapy in the context of the underlying immunodeficiency and recurrent infections rather than as treatment for immune thrombocytopenia. Systemic corticosteroid therapy was initiated at 2 mg/kg/day of prednisone due to severe thrombocytopenia in the setting of an initially unconfirmed diagnosis of WAS, with subsequent gradual tapering according to clinical and hematological response. Cyclosporine was introduced at an initial dose of 3 mg/kg/day in two divided doses, with dose adjustment according to clinical response. Cyclosporine was later discontinued because of poor adherence, mainly related to financial constraints and limited access to treatment. Rituximab was not considered, as the patient did not develop autoimmune manifestations. Eltrombopag was not available in our setting during follow-up. Hematopoietic stem cell transplantation was not feasible because of the absence of a suitable HLA-matched donor and financial constraints.

At the age of four years, the patient developed a severe neurological complication consisting of a left frontoparietal subdural hematoma secondary to severe thrombocytopenia, presenting with signs of intracranial hypertension, including severe headache and vomiting (Figure 3). Brain imaging showed no significant mass effect. Medical management was undertaken, including corticosteroid therapy, platelet transfusions, and prophylactic antiepileptic treatment with valproic acid, with no indication for neurosurgical intervention. The clinical outcome was favorable, with progressive clinical recovery, no recurrence of intracranial hemorrhage, and no apparent neurological sequelae during follow-up.

**Figure 3:**
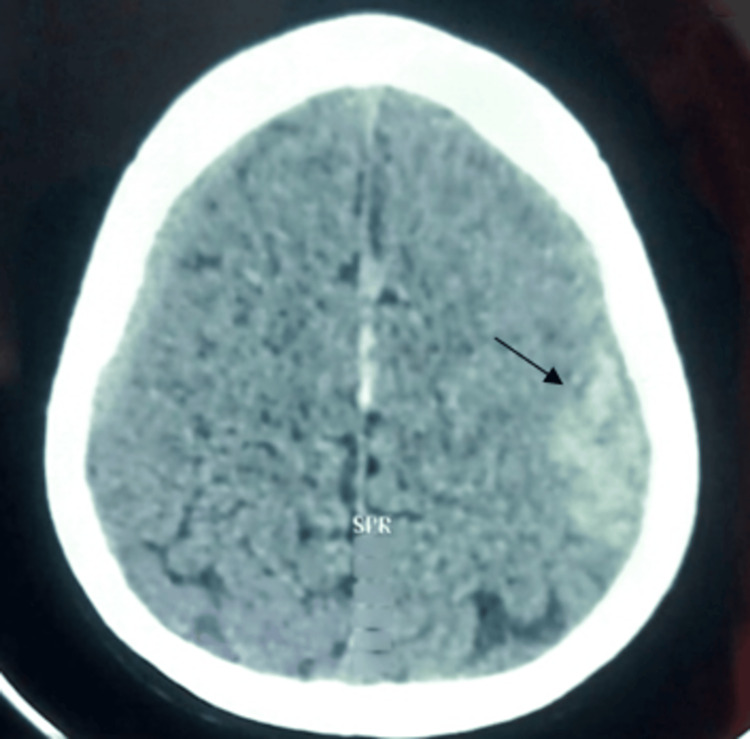
Brain CT scan showing intracranial hematoma Non-contrast brain computed tomography (CT) scan demonstrating an intracranial hematoma (arrow), consistent with a severe hemorrhagic complication in the context of Wiskott-Aldrich syndrome-associated thrombocytopenia.

Follow-up also revealed the recent onset of a similar clinical presentation in a four-month-old younger brother, characterized by purpura and peripheral thrombocytopenia, currently under evaluation within the same etiological framework.

The patient is currently five years old and continues to be followed for genetically confirmed WAS, with persistent thrombocytopenia and recurrent mucocutaneous bleeding episodes. Regular complete blood count monitoring is performed. Platelet transfusions are administered only when clinically indicated because of severe thrombocytopenia or significant hemorrhagic manifestations, given the potential risk of alloimmunization and its possible impact on future hematopoietic stem cell transplantation. Long-term surveillance is maintained because of the recognized risk of autoimmune and malignant complications associated with WAS.

## Discussion

First described in 1937 by Wiskott in three brothers presenting with thrombocytopenia, eczema, recurrent infections, and early fatal hemorrhagic and infectious complications, WAS was later confirmed as an X-linked disorder by Aldrich et al. in 1954 [4,5]. It is now defined as a rare X-linked primary immunodeficiency, with an estimated incidence of one to four cases per 1,000,000 live male births, caused by mutations in the WAS gene leading to deficiency of WAS protein (WASp) and resulting in combined immune and platelet dysfunction [3,6], characterized by the classical triad of immunodeficiency, eczema, and thrombocytopenia, and a broad clinical spectrum including autoimmune and malignant complications [2].

Clinical manifestations of WAS are highly variable, ranging from classical severe disease to milder phenotypes such as X-linked thrombocytopenia, depending on the underlying mutation and residual WASp expression [2]. Although microthrombocytopenia is classically considered a hallmark, atypical presentations with normal or variable platelet size have been reported, highlighting the phenotypic variability [7,8]. In our patient, the peripheral blood smear showed platelet anisocytosis without microplatelets despite severe thrombocytopenia. The diagnosis of WAS relies on molecular analysis of the WAS gene, which remains the gold standard, while flow cytometric assessment of WASp expression provides a useful screening tool [3]. In our case, the diagnosis was strongly suspected based on the clinical triad of early-onset eczema, recurrent infections, and persistent thrombocytopenia, and was subsequently confirmed genetically, despite the absence of classical microthrombocytopenia. Overall, these observations, together with previously reported cases, suggest that platelet morphology may be more variable than initially described, and that diagnosis should rely on a combination of clinical, biological, and molecular findings [7,8].

WAS can be graded using a severity score (0-5) based on thrombocytopenia, eczema, immunodeficiency, and autoimmune or malignant manifestations, with classical forms typically scoring ≥3, severe forms reaching 5, and milder phenotypes such as X-linked thrombocytopenia around 2 [3,9]. This classification reflects a dynamic clinical spectrum and helps guide long-term management and follow-up. In our case, the presentation was consistent with classical WAS, with severe persistent thrombocytopenia, early-onset eczema, recurrent infections, and bleeding manifestations, corresponding to a score ≥3. Although no autoimmune or malignant complications were observed, the occurrence of intracranial hemorrhage highlights the severity of hematological involvement and the need for close follow-up. 

In the absence of appropriate care and timely intervention, morbidity and mortality remain high in WAS, with reported deaths in non-transplant patients mainly due to infections, hemorrhages, and malignancies [5,10]. Hemorrhagic complications, driven by severe thrombocytopenia and vascular fragility, represent a major cause of mortality. Intracranial hemorrhage, although rare, is particularly severe and potentially fatal, with reported cases including that of Manral et al. In our case, the patient developed a subdural hematoma in the setting of profound thrombocytopenia; however, early management resulted in a favorable outcome [11].

The management of WAS is multidisciplinary and aims to prevent and treat infectious, hemorrhagic, and immune complications. Intravenous immunoglobulin (IVIG) provides replacement and immunomodulatory effects but does not correct thrombocytopenia [12], while antibiotic prophylaxis (e.g., cotrimoxazole) is recommended to prevent opportunistic infections [12]. Platelet transfusions remain essential in severe bleeding episodes despite the risk of alloimmunization [3]. Splenectomy may improve thrombocytopenia in selected cases but is limited by infection risk and implications for future hematopoietic stem cell transplantation [13]. Immunosuppressive agents such as corticosteroids and cyclosporine may provide symptomatic benefit in autoimmune or refractory cytopenias [3]. In our patient, management was mainly supportive with IVIG and antibiotic prophylaxis, with persistent severe thrombocytopenia requiring transfusions. Cyclosporine adherence was suboptimal, and eltrombopag, which could represent a potentially effective option for refractory thrombocytopenia, was not available at the time of management.

Hematopoietic stem cell transplantation (HSCT) remains the only curative treatment for WAS and should be considered early, as younger age at transplantation is associated with better outcomes, particularly before five years, even with unrelated donors [3,13]. In the absence of an HLA-identical related donor, an unrelated donor search is recommended, especially in severe forms, whereas HSCT is generally avoided in isolated thrombocytopenia without a matched donor. Neonatal and infant data remain limited [3,13]. In our case, the patient has not yet undergone HSCT, underscoring the importance of early referral and systematic evaluation for transplantation.

## Conclusions

This case highlights an atypical presentation of genetically confirmed WAS characterized by severe thrombocytopenia without microthrombocytopenia and complicated by intracranial hemorrhage. It emphasizes that normal platelet size should not exclude the diagnosis of WAS and supports the use of molecular testing in clinically suggestive cases. Early recognition, appropriate supportive management, and timely evaluation for hematopoietic stem cell transplantation remain essential to improve patient outcomes.

## References

[1] Bousfiha AA, Jeddane L, Moundir A (2025). The 2024 update of IUIS phenotypic classification of human inborn errors of immunity. J Hum Immun.

[2] Ochs HD, Filipovich AH, Veys P, Cowan MJ, Kapoor N (2009). Wiskott-Aldrich syndrome: diagnosis, clinical and laboratory manifestations, and treatment. Biol Blood Marrow Transplant.

[3] Buchbinder D, Nugent DJ, Fillipovich AH (2014). Wiskott-Aldrich syndrome: diagnosis, current management, and emerging treatments. Appl Clin Genet.

[4] Wiskott A (1937). Familial, congenital Werlhof's disease? [Article in German]. Monatsschr Kinderheilkd.

[5] Aldrich RA, Steinberg AG, Campbell DC (1954). Pedigree demonstrating a sex-linked recessive condition characterized by draining ears, eczematoid dermatitis and bloody diarrhea. Pediatrics.

[6] Huynh PN, Kali MA (2024). Wiskott-Aldrich syndrome. https://emedicine.medscape.com/article/137015-overview.

[7] Mantadakis E, Sawalle-Belohradsky J, Tzanoudaki M, Kanariou M, Chatzimichael A, Albert MH (2014). X-linked thrombocytopenia in three males with normal sized platelets due to novel WAS gene mutations. Pediatr Blood Cancer.

[8] Mawalla WF, Iddy H, Kindole CA, Nasser A, Schuh A (2021). Wiskott-Aldrich syndrome with normal platelet volume in a low-income setting: a case report. Ther Adv Rare Dis.

[9] Mahlaoui N, Pellier I, Mignot C (2013). Characteristics and outcome of early-onset, severe forms of Wiskott-Aldrich syndrome. Blood.

[10] Sullivan KE, Mullen CA, Blaese RM (1994). A multiinstitutional survey of the Wiskott-Aldrich syndrome. J Pediatr.

[11] Manral I, Sagoo GK, T A, Manral R, Radhakrishna KV, Makhani CS, Khan RN (2025). Disease, accident, and trauma: a case report on sudden death in Wiskott-Aldrich's syndrome. Acad Forensic Pathol.

[12] Litzman J, Jones A, Hann I, Chapel H, Strobel S, Morgan G (1996). Intravenous immunoglobulin, splenectomy, and antibiotic prophylaxis in Wiskott-Aldrich syndrome. Arch Dis Child.

[13] Ozsahin H, Cavazzana-Calvo M, Notarangelo LD (2008). Long-term outcome following hematopoietic stem-cell transplantation in Wiskott-Aldrich syndrome: collaborative study of the European Society for Immunodeficiencies and European Group for Blood and Marrow Transplantation. Blood.

